# Single- versus Multiple-Tract Percutaneous Nephrolithotomy in the Surgical Management of Staghorn Stones or Complex Caliceal Calculi: A Systematic Review and Meta-analysis

**DOI:** 10.1155/2020/8817070

**Published:** 2020-12-17

**Authors:** Binbin Jiao, Zhenshan Ding, Zhenkai Luo, Shicong Lai, Xin Xu, Xing Chen, Guan Zhang

**Affiliations:** ^1^Graduate School of Peking Union Medical College and Chinese Academy of Medical Sciences, Beijing, China 100029; ^2^Department of Urology, China-Japan Friendship Hospital, Yinghuadong Road, Chaoyang District, Beijing 100029, China; ^3^Peking University China-Japan Friendship School of Clinical Medicine, Yinghuadong Road, Chaoyang District, Beijing 100029, China; ^4^Department of Urology, Beijing Hospital, No. 1, Dahua Road, Dongdan, Dongcheng District, Beijing 100005, China

## Abstract

**Objective:**

To assess current evidence on the effectiveness and safety of single- versus multiple-tract percutaneous nephrolithotomy in the surgical management of complex caliceal calculi or staghorn stones through a comprehensive literature review.

**Methods:**

A comprehensive literature review of articles investigating the clinical efficacy and safety of single- versus multiple-tract percutaneous nephrolithotomy was performed. Relevant literature was obtained by systematically searching PubMed, EMBASE, and the Cochrane Library through May 2020. We followed the search strategy based on the Preferred Reporting Items for Systematic Reviews and Meta-Analyses statement. The primary outcomes, including the stone-free rate (SFR), and secondary outcomes (peri- and postoperative complications and operative data) were evaluated using RevMan 5.3 statistical software.

**Results:**

Ten studies involving 1844 patients with complex caliceal calculi or staghorn stones met the inclusion criteria. Single-tract percutaneous nephrolithotomy (STPCNL) had noninferior clinical efficacy with respect to the immediate SFR (odds ratio (OR) = 0.80, 95% confidence interval (CI) (0.46 to 1.38), *p* = 0.42) and 3-month SFR (OR = 1.22, 95% CI (0.38 to 3.92), *p* = 0.74) compared with multiple-tract percutaneous nephrolithotomy (MTPCNL). In addition, pooled analyses showed that STPCNL resulted in significantly lower hemoglobin decreases (MD = −0.46, 95% CI (-0.68 to -0.25), *p* < 0.0001), fewer blood transfusions (OR = 0.48, 95% CI (0.34 to 0.67), *p* < 0.0001), and fewer pulmonary complications (OR = 0.28, 95% CI (0.09 to 0.83), *p* = 0.02) than MTPCNL. However, the overall evidence was insufficient to suggest a statistically significant difference for other adverse events.

**Conclusion:**

This meta-analysis indicated that STPCNL is an effective method for treating complex caliceal calculi or staghorn stones. Compared with MTPCNL, STPCNL not only yields similarly high SFRs but also is associated with many advantages, less blood loss, fewer blood transfusions, and fewer pulmonary complications without an increase in other complications. However, the findings of this study should be further confirmed by well-designed prospective randomized controlled trials (RCTs) with a larger patient series.

## 1. Introduction

Ureteral calculi represent a common disease that seriously endangers life and work for more than 12% of the population. Staghorn or complex caliceal calculi constitute one of the most challenging problems in urology and are likely to destroy the function of the kidney and cause life-threatening sepsis [1]. For patients with staghorn or complex caliceal calculi, the goal of treatment is to achieve maximal clearance of stones and assure maximal renal function preservation with minimal complications. In the recently updated guidelines of the American Urological Association Nephrolithiasis Guideline Panel on Staghorn Calculi, percutaneous nephrolithotomy (PCNL) is an integral component of the management of most staghorn and large-volume renal calculi [2]. However, complex caliceal and staghorn stones are difficult to remove with a single-tract PCNL approach [3]. A trend toward the use of percutaneous monotherapy using multiple tracts as the preferred treatment option for most staghorn or complex calculi has emerged [4]. However, a concern with creating multiple percutaneous tracts is the potential risks of greater bleeding and higher complication rates compared with the single-tract approach [5]. Therefore, many urologists hesitate to place multiple percutaneous tracts during PCNL. In recent years, an increasing number of studies have been conducted to assess the clinical efficacy, operative results, and complications of STPCNL and MTPCNL, but the outcomes of these studies have varied. Thus, conducting a new systematic review and meta-analysis that includes relevant available studies evaluating the efficacy of single- versus multiple-tract PCNL in the surgical management of complex caliceal calculi or staghorn stones is worthwhile.

## 2. Method

### 2.1. Search Strategy

To assess the clinical efficacy and safety of single- versus multiple-tract PCNL, a comprehensive literature search was performed using PubMed, EMBASE, and the Cochrane Library in February 2020. We considered the definition of staghorn calculi to be stones that branched and occupied a large portion of the collecting system, such as complete staghorn stones (occupying the renal pelvis and the entire caliceal system or occupying 80% of the renal collecting system) or partial staghorn stones (occupying the renal pelvis or at least two or more calices), and we defined complex caliceal calculi as those with a large bulk and involving more than one calix, the upper ureter, or both. The keywords “single tract”, “multiple tract”, “percutaneous nephrolithotomy”, “complex caliceal calculi”, and “staghorn stones” were used to search for articles. These search terms were used individually and in combination. Additionally, manual searches of the references and citation lists of all relevant reviews were performed. For publication selection, a search strategy was applied based on the Preferred Reporting Items for Systematic Reviews and Meta-Analyses (PRISMA) statement and the assessing the methodological quality of systematic reviews (AMSTAR) guidelines.

Studies meeting the following criteria were included: (1) studies comparing the safety and efficacy of single- versus multiple-tract PCNL for surgical treatment of complex caliceal calculi or staghorn stones; (2) outcome measures consisting of at least one of the following treatment-related adverse events and functional outcomes: the stone-free rate (SFR), complications, hospitalization times, operative times, blood loss, and blood transfusion when available; and (3) articles written in English with the full text or related data available. The exclusion criteria were as follows: (1) duplicate publications or conference proceedings; (2) nonpublished materials, editorials, or reviews; and (3) studies containing patients with serious urinary infection, renal insufficiency, musculoskeletal deformities, solitary kidney, or congenital abnormalities.

**Figure 1:**
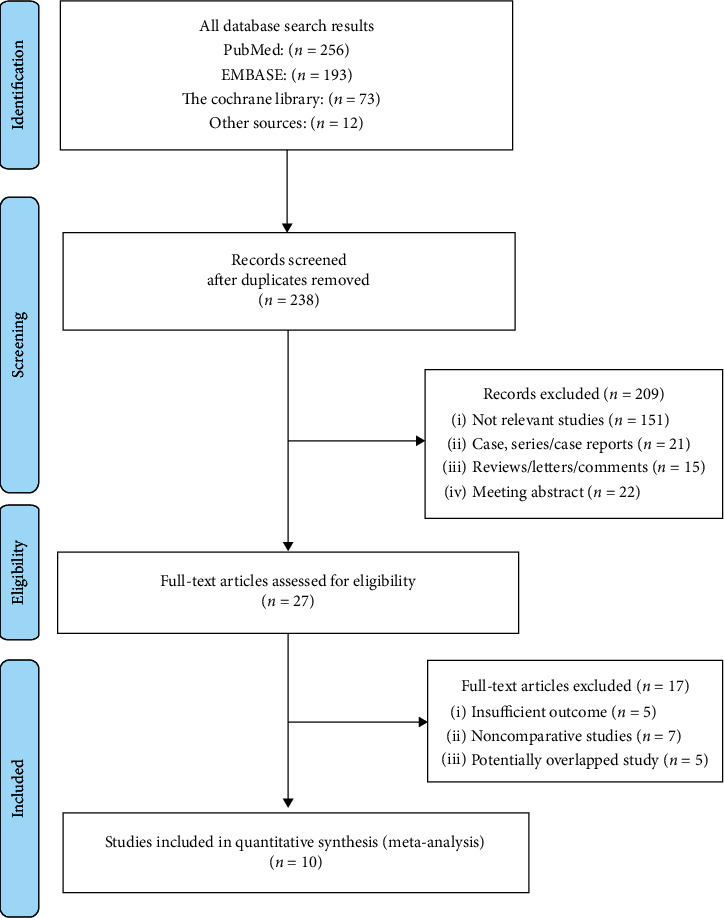
Study selection flowchart.

Relevant references cited in the selected papers were also retrieved. The literature search and selection were independently performed by 3 reviewers (J.B., D.Z., and Z.L.) and then cross-checked. Any differences at this stage were resolved through discussion and by a majority decision of the reviewers if necessary. A flowchart showing the number of publications selected or excluded at each stage is presented in Figure 1. Ethics committee approval for this study was not necessary because all the data were carefully extracted from the existing literature, and this article did not involve individual patient data.

### 2.2. Assessment of Study Quality

We evaluated the level of evidence for each selected article based on the criteria recommended by the Oxford Centre for Evidence-based Medicine [6]. For the methodological quality assessment, we used the Newcastle-Ottawa Scale (NOS) [7] to evaluate the quality of prospective studies. In addition, we evaluated the methodological quality of the trials according to the methods recommended by the Cochrane Collaboration.

Preoperative parameters were extracted together with intraoperative data, including operation times, hemoglobin decreases, and transfusion rates. Postoperative data, including the SFR, length of hospitalization, and treatment-related complications, were also analyzed. Functional results, including renal function, were assessed after surgery.

Ten relevant studies [8–17] including 1844 patients were selected for analysis. No differences were found in terms of age and basic physical conditions between the MTPCNL and STPCNL groups. Data extraction was independently performed by 2 reviewers (J.B. and Z.L.) and then cross-checked. Any differences at this stage were resolved through discussion and by a majority decision of the reviewers if necessary.

We used the mean difference (MD) to evaluate continuous outcomes. For the studies expressing continuous data as the median and range values, we used the statistical formula described by Wan et al. [18] to determine the mean and standard deviation in accordance with the recommended methods described in the Cochrane Handbook for Systematic Reviews 23.

The results are expressed as the odds ratio (OR) with a 95% confidence interval (CI) for dichotomous variables. The *χ*^2^ and *I*^2^ tests were used to assess the heterogeneity of the study data (*I*^2^ > 50% was regarded as substantial heterogeneity). If the heterogeneity was considered low, fixed effects models were used for the meta-analyses. Otherwise, a random effects model was used to reduce the effect of statistical heterogeneity. The pooled effects were determined by a *z* test, and a *p* value < 0.05 was considered statistically significant. Moreover, for the comparisons of MTPCNL and STPCNL, relevant publications with appropriate data allowed us to perform subgroup analyses according to the device used. For several comparisons, sensitivity analyses were used. The meta-analysis of comparable data was performed using Review Manager (RevMan) 5.3 software.

## 3. Results

The initial search strategy yielded 534 studies from the metadatabases combined. Our strict eligibility criteria resulted in the exclusion of 524 reports. Ten studies focusing on two different complex caliceal calculus or staghorn stone interventions were included, which involved 1844 participants, 48.26% (*n* = 890) of whom underwent STPCNL, while 51.73% (*n* = 954) underwent MTPCNL. Examination of the references listed for these studies and for the review articles did not yield any further studies for evaluation.

### 3.1. Characteristics of the Selected Studies

Patient characteristics and study characteristics are summarized in Table 1. No differences were found in basic physical conditions for all of the included studies. The outcome parameters for the differential management of complex caliceal calculi or staghorn stones are shown in Table 2.

### 3.2. SFR

**Figure 2:**
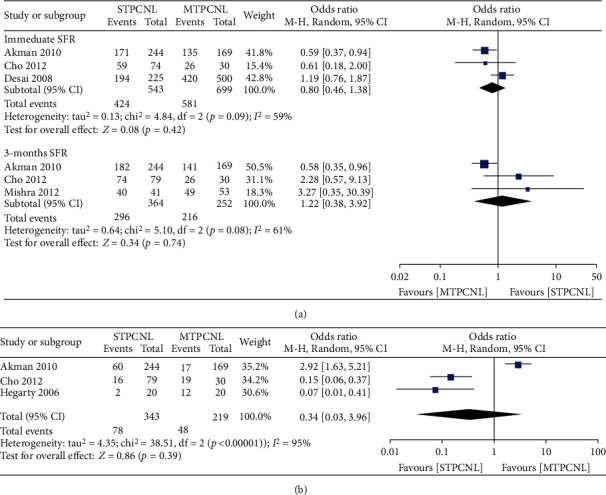
Forest plots comparing (a) immediate SFR and 3-month SFR and (b) auxiliary treatment.

Three included studies compared the immediate SFRs of single- versus multiple-tract PCNL. The random effects model was selected for analysis due to significant heterogeneity among these trials (*I*^2^ = 59%). The overall results showed no significant difference between single- and multiple-tract PCNL for the immediate SFR (OR = 0.80, 95% CI (0.46 to 1.38), *p* = 0.42) (Figure 2(a)). In addition, a random effects model was used to analyze the 3-month SFR. No significant difference was found between the single- and multiple-tract PCNL SFRs (OR = 1.22, 95% CI (0.38 to 3.92), *p* = 0.74) (Figure 2(a)). The sensitivity analysis suggested the same results.

### 3.3. Auxiliary Treatment

Auxiliary procedures to achieve stone-free status included shockwave lithotripsy, “sandwich therapy,” repeat PCNL, and URS after STPCNL or MTPCNL. The use of such procedures was reported in two studies focusing on STPCNL and MTPCNL. A pooled analysis showed no significant difference between the two groups for repeat or auxiliary treatment (OR = 0.34, 95% CI (0.03-3.96), *p* = 0.39) (Figure 2(b)).

### 3.4. Hemoglobin Decreases

**Figure 3:**
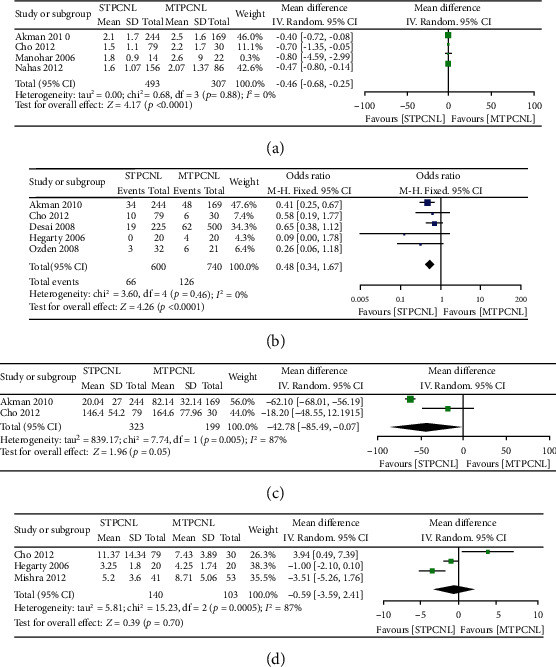
Forest plots comparing (a) hemoglobin decreases, (b) blood transfusion, (c) operation time, and (d) hospitalization time.

Four studies reporting operative hemoglobin decreases and comparing STPCNL to MTPCNL were included in this meta-analysis, and a significantly lower operative hemoglobin decrease was observed for STPCNL than MTPCNL (MD = −0.46, 95% CI (-0.68 to -0.25), *p* < 0.0001) (Figure 3(a)).

### 3.5. Blood Transfusion

Very few blood transfusion events were reported in five studies comparing single- versus multiple-tract PCNL. A meta-analysis by the fixed effects model (*I*^2^ = 0%) demonstrated a statistical difference between single- and multiple-tract PCNL with respect to blood transfusion (OR = 0.48, 95% CI (0.34 to 0.67), *p* < 0.0001) (Figure 3(b)).

### 3.6. Operative Time

Meta-analysis by the random effects model (*I*^2^ = 87%) demonstrated no significant difference between STPCNL and MTPCNL with respect to the operative time (MD = −42.78 min, 95% CI (0-85.49 to -0.07), *p* = 0.05) (Figure 3(c)).

### 3.7. Hospitalization Time

Regarding the length of inpatient stay, three studies were included in this meta-analysis. When pooled, the overall result showed that the STPCNL group and the MTPCNL group were similar with regard to this outcome (MD = −0.59, 95% CI (-3.59 to 2.41), *p* = 0.70) (Figure 3(d)).

### 3.8. Renal Function

**Figure 4:**
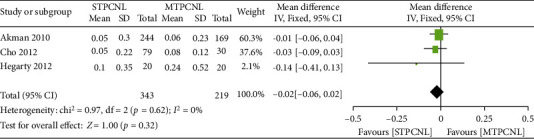
Forest plots of renal function.

Regarding serum creatinine, three studies comparing STPCNL to MTPCNL were included. On the basis of our analysis, no heterogeneity was found among the trials (*I*^2^ = 0); thus, a fixed effects model was selected. The meta-analysis showed no difference between STPCNL and MTPCNL (MD = −0.02, 95% CI (-0.06 to 0.02), *p* = 0.32) (Figure 4).

### 3.9. Complications

**Figure 5:**
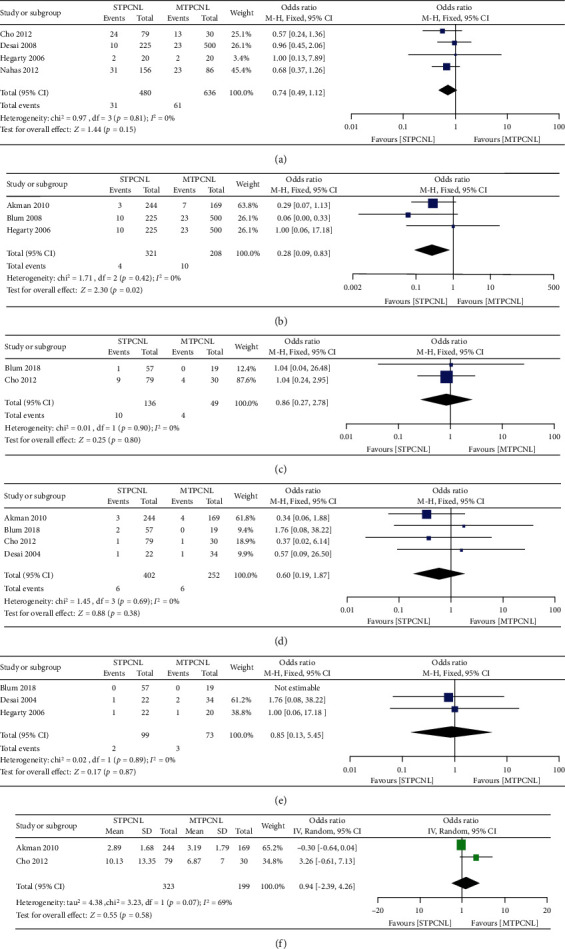
Forest plots of other parameters including (a) complications, (b) pulmonary complications, (c) postoperative fever, (d) urine leakage, (e) urinary tract infection, (f) nephrostomy time.

Overall complications were reported in 4 studies. A total of 128 events were reported among 1116 participants. On the basis of our analysis, no heterogeneity was found among the trials (*I*^2^ = 0). The pooled analysis revealed no significant difference in the incidence of postoperative fever between the two groups (OR = 0.74, 95% CI (0.49 to 1.12), *p* = 0.15) (Figure 5(a)). However, the combined overall result showed that STPCNL resulted in a lower risk of pulmonary complications (OR = 0.28, 95% CI (0.09–0.83), *p* = 0.02) (Figure 5(b)). The meta-analyses detected no significant differences in other complications, such as fever (OR = 0.86, 95% CI (0.27 to 2.78) (Figure 5(c)), *p* = 0.80), urine leakage (OR = 0.6, 95% CI (0.19 to 1.87), *p* = 0.38) (Figure 5(d)), urinary tract infection (OR = 0.85, 95% CI (0.13 to 5.45), *p* = 0.87) (Figure 5(e)), and nephrostomy time (MD = 0.94, 95% CI (-2.39 to 4.26), *p* = 0.58) (Figure 5(f)).

### 3.10. Publication Bias

**Figure 6:**
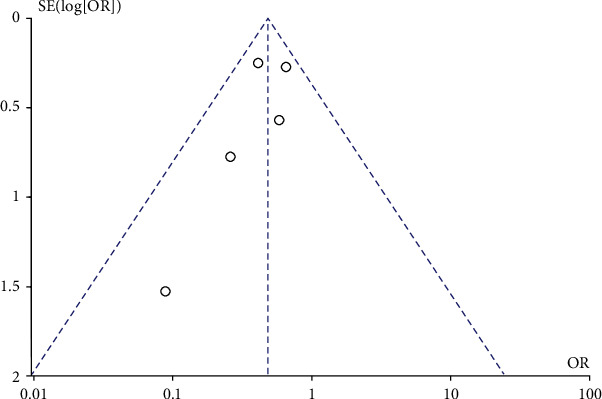
Funnel plot for evaluation of potential publication bias.

A funnel plot was generated to assess publication bias (Figure 6). The result showed no apparent asymmetry, which indicated no obvious publication bias.

## 4. Discussion

Kidney calculi are a common urological disorder characterized by a high recurrence rate. Staghorn stones or complex caliceal calculi still represent an intractable challenge for urologists. PCNL is an integral component of the management of most staghorn and large-volume renal calculi [19]. The recently updated guidelines by the American Urological Association panel on staghorn calculi recommend the use of percutaneous monotherapy using multiple tracts as the preferred treatment option for most staghorn calculi. Although the safety of creating percutaneous renal tracts is well established, a concern regarding the use of multiple tracts remains due to additional complications compared to STPCNL. Although many studies have shown the effectiveness of single- versus multiple-tract PCNL for the treatment of complex caliceal calculi or staghorn stones, the results have been controversial. The purpose of this meta-analysis was to evaluate and compare the efficacy and safety of single- versus multiple-tract PCNL in the surgical management of complex caliceal calculi or staghorn stones.

The SFR is the most important parameter for estimating the clinical efficacy of surgical methods for stones. In this meta-analysis, the pooled analysis revealed no significant difference in the SFR between the STPCNL and MTPCNL groups, indicating that STPCNL is an effective method for treating staghorn or complex caliceal calculi. However, many studies have shown the differential effectiveness of STPCNL and MTPCNL for the treatment of staghorn or complex caliceal calculi. After consulting relative literatures, the different results for the SFRs may be associated with the following factors. First, endourological societies have not agreed on a clear definition of the SFR, which has been defined as the presence of residual stones ranging from 0 to 4 mm in size. Second, SFRs may have been different if different tools were used for postoperative assessments. Some studies preferred to assess SFRs by KUB or ultrasound to identify clinically significant residual fragments while reducing radiation exposure to the patient [9]. However, evaluating the SFR using CT is more accurate as CT can better detect smaller residual stones [20]. In most of the literature, PCNL was primarily used as a part of combination therapy when managing staghorn or complex calculi [10]. Moreover, some studies have reported that the SFRs after PCNL monotherapy for staghorn stones ranged from 49% to 78% [21, 22]. Streem et al. [23] reported an SFR of 63–70% when they used “sandwich therapy” with extracorporeal shockwave lithotripsy (ESWL), where PCNL was the terminal procedure (PCNL–ESWL–PCNL). In addition, several sessions of PCNL may be necessary to remove all stone branches [24], and repeat or auxiliary treatment such as ESWL might be required for residual fragments to ensure the SFR. The SFR is also related to the follow-up time as the final SFR after surgery is higher than that immediate SFR after surgery. Time is needed for stone fragments to be flushed out with urine. Some studies involved treatment for complete staghorn stones, while other studies included partial and complete staghorn stones. Unfortunately, we were unable to conduct a subgroup analysis to analyze the influence of the number of tracts on treatment effectiveness due to insufficient data. Last, certain points of surgical techniques merit special emphasis regarding the SFR. Renal access should be established by endourologists with considerable experience in percutaneous surgery because they will be most familiar with the pelvicalyceal anatomy and the surgical procedure. Accordingly, more studies are needed to obtain more reliable outcomes.

Major complications of PCNL include bleeding. The results of this meta-analysis showed that the use of multiple tracts contributed to a higher hemoglobin drop and a higher frequency of transfusions. These results are consistent with previously published results. Hemorrhage is generally associated with the initial puncture and injury of renal blood vessels and the surrounding organs. Additional tracts during PCNL may increase the risk of injury to major blood vessels and may complicate recovery from puncture injury. In this meta-analysis, we analyzed only the overall blood loss and transfusion events, but we did not analyze subgroups for the number of tracts due to the low amount of data from the included studies due to insufficient data. However, in some studies, multiple tracts did not significantly increase blood loss and transfusion requirements. Hegarty and Desai noted a mean drop in hemoglobin in patients with multiple tracts similar to that in patients with single tracts [12, 14, 25]. They thought that this result was probably related to lower baseline hemoglobin concentrations, and transfusions were performed on the second or third postoperative day rather than as an emergency for significant blood loss. Although an increase in the number of tracts has adverse effects in terms of blood loss, many measures can be implemented to avoid more blood loss. First, direct puncture into the pelvis or near the infundibular neck may be avoided to reduce the risk of severe bleeding [26]. Second, selecting the size of the tract based on the width of the funnel and the angle at which the tract enters the renal pelvis may help prevent the overdilatation of the infundibulum and subsequent significant bleeding [15]. In addition, staging the procedure may strengthen the urethra, rendering PCNL surgery relatively simple without massive blood loss [5]. Furthermore, based on our experience, we suggest using gentle techniques, avoiding manipulations such as levering the nephroscope and using flexible endoscopes and nitinol baskets when necessary to prevent bleeding complications. Finally, some doctors have suggested that the use of balloon dilators was associated with lower blood loss. The use of Amplatz dilators may be a reason for relatively high blood loss.

For complex or multiple stones, more than one percutaneous access may be required for stone disintegration, potentially increasing the risk of parenchymal injury and reducing kidney function [27]. Although we found no significant difference in renal function based on serum creatinine, a trend toward MTPCNL being associated with a greater reduction in renal function was noted. Some authors have found that multiaccess PCNL was associated with a significant decrease in the renal function of the targeted kidney according to MAG3 nuclear renogram results compared to a single-tract approach [13]. Therefore, utilizing a minimal number or smaller PCNL tracts that achieve optimal stone removal with the least degree of injury to the renal parenchyma is advisable. Although patients undergoing MTPCNL may demonstrate a postoperative decline in renal function, some studies have noted that this change was temporary and reversible. The authors indicated a statistically significant improvement based on the glomerular filtration rate (eGFR) at the first postoperative year, which worsened in only 6.8% of patients with a solitary kidney who had been treated with PCNL [8]. Some authors thought that increases in creatinine levels at an early postoperative period were temporary and probably associated with anesthetic agents and medications [14, 25]. Patients with stone disease and baseline renal insufficiency ultimately show improvements in renal function as a result of the relief of obstruction and resolution of infection with complete calculus clearance [5, 28]. Numerous studies have found no deleterious effects of PCNL on renal function among many patients, including multiple-access procedures [11, 29, 30]. However, renal function is generally assessed with different methods, such as the eGFR or serum creatinine, the efficacy of which can be substantially influenced by patient factors, including body mass index (BMI), baseline renal function, and ethnicity [31–33]. Furthermore, the eGFR may serve as an estimate of global nephron function but not for unilateral kidney PCNL; only a single kidney unit is affected. Recently, nuclear renography using the radioisotope technetium-99m mercaptoacetyltriglycine (99mTc-MAG3) has been commonly used to assess urinary obstruction as well as the relative percent function of each kidney. One of the limitations of this meta-analysis was the small number of studies with detailed data for more than two tracts. In fact, most multiple-tract PCNLs had only two access tracts. Thus, we cannot conduct a subgroup analysis to analyze the number of access tracts and the influence of the number of tracts on renal function.

When comparing morbidity between single and multiple tracts, the blood transfusion rate was higher in patients with multiple tracts, but most complications were similar and not significantly different between groups in our meta-analysis. However, we found that multiple-tract PCNL may lead to higher rates of pulmonary complications. Upper-pole (UP) access is well documented to be associated with a higher risk of thoracic complications such as pneumothorax and hydrothorax, especially with punctures that originate above the 11th rib [34–36]. Pulmonary injury due to lung transgression can occur even with controlled expirations anywhere from 14% on the left side to 29% on the right side [37, 38]. The rate of pulmonary complications is unequivocally higher with supracostal UP approaches, with some sources describing occurrence rates greater than 15% [39]. Despite this finding, the literature tends to support the continued use of UP access citing more expeditious, direct, and complete stone removal with fewer access sites [9]. After reviewing many studies, many measures can reduce the rates of complications. First, for supracostal UP access, the puncture site can be localized in the midpoint of the 11th and 12th intercostal spaces, usually 1 to 2 cm cranial to the upper pole of the most medial calix [8]. The advantages of direct UP access are good exposure to most of the calices and the renal pelvis, the possibility of reaching the ureteropelvic junction and upper ureter, and the ability to operate along the long axis of the kidney, which causes less torque of the rigid nephroscope and ultimately less bleeding. In our experience, access through a lower calix into the upper and middle calix is difficult and necessitates longer operative and fluoroscopy times. Second, when deciding to use MTPCNL, placing all the tracts and fixing all guidewires before starting dilatation of the first tract are advisable [17]. In addition, preoperative planning of the procedure and selecting the appropriate technique must be individualized for each patient. On the basis of our assessment of calculus configuration and collecting system anatomy, all possible percutaneous tracts were punctured right at the outset, and guidewires were secured because confirming correct percutaneous needle placement is significantly easier in an intact collecting system [10].

To the best of our knowledge, we are the first to evaluate the current evidence on the effectiveness and safety of single- versus multiple-tract PCNL in the surgical management of complex caliceal calculi and staghorn stones following the PRISMA guidelines. However, limitations should be considered when drawing conclusions. First, because most of the included studies were retrospective, biases from the original studies, such as selection bias, information bias, and other confounding factors, likely have not been excluded in this meta-analysis. Although all ten eligible studies involving 1844 patients were of moderate quality (scores ≥ 7) according to the NOS, bias still exists, which might render these results less reliable.

Second, in addition to the surgical process, other important clinical parameters, such as stone size, complete or incomplete staghorn stones, stone location, and stone composition, were important factors influencing effectiveness and safety. Therefore, conducting subgroup analyses to compare the efficacy of these two approaches may have rendered the findings more generalizable. Unfortunately, we were unable to conduct a subgroup analysis to analyze the influence of the number of tracts on effectiveness due to insufficient data. Therefore, we hope that intercalating additional data will shed light on these outcomes in the future.

Finally, despite the well-recognized advantages of meta-analyses, the results were predictably affected by the quality of the included studies and reporting biases, which may have occurred due to the lack of studies confirming the null hypothesis or publishing nonsignificant outcomes. Studies reporting nonsignificant outcomes have historically been more difficult to publish than studies showing statistically significant results. One would hope that as the publication process matures, this evidence base will become more sophisticated, which may limit the influence of publication bias. Given these limitations, we hope that well-designed prospective trials are designed to verify the findings of this meta-analysis in the future.

Despite these limitations, this study represents the first meta-analysis comparing STPCNL and MTPCNL in the surgical management of staghorn or complex caliceal calculi. Thus, we provide the most up-to-date information on the surgical treatment of patients with complex caliceal calculi or staghorn stones, which we hope can provide some help to urologists and patients when selecting the optimal therapy. However, the findings of this study should be further confirmed by well-designed prospective randomized controlled trials (RCTs) with a larger patient series.

## 5. Conclusion

Our systematic review and meta-analysis demonstrated that STPCNL seems to be a safe and feasible alternative compared to MTPCNL for patients with staghorn stones and has many advantages, such as no decreases in the final SFR, fewer blood transfusions, and even fewer complications, such as pulmonary complications and postoperative fever. However, our conclusion should be treated prudently, and further large-sample, prospective, and multicenter studies and RCTs should be undertaken to confirm our findings.

## Figures and Tables

**Table 1:** Summary of comparative studies included in meta-analysis.

Study	Country	Study period	Study design	LE	Intervention		Sample size		Study quality
					Trial	Control	Trial	Control	
Blum 2018	America	Not mentioned	Prospective study	2b	STPCNL	MTPCNL	57	19	8^#^
Nahas 2012	Egypt	1999-2009	Not mentioned	2b	STPCNL	MTPCNL	156	86	7^#^
Mishra 2012	India	2009-2010	Retrospective study	2a	STPCNL	MTPCNL	41	53	7^#^
Akman 2010	Turkey	2002-2009	Retrospective study	2a	STPCNL	MTPCNL	244	169	7^#^
Hegarty 2006	America	2004-2005	Retrospective study	2a	STPCNL	MTPCNL	20	20	7^#^
Desai 2004	India	1991-2002	Retrospective study	2b	STPCNL	MTPCNL	22	34	6^#^
Cho 2012	Korea	2003-2008	Not mentioned	2b	STPCNL	MTPCNL	79	30	7^#^
Ozden 2008	Turkey	2000–2005	Retrospective study	2b	STPCNL	MTPCNL	32	21	7^#^
Desai 2008	India	1991-2007	Retrospective study	2b	STPCNL	MTPCNL	225	500	8^#^
Manohar 2006	India	1991-2004	Not mentioned	2a	STPCNL	MTPCNL	14	22	7^#^

LE = level of evidence; STPCNL = single-tract percutaneous nephrolithotomy; MTPCNL = multiple-tract percutaneous nephrolithotomy. ^#^Using Newcastle-Ottawa Scale (score from 0 to 9).

**Table 2:** Study outcomes comparing STPCNL and MTPCNL.

Outcomes	No. of studies	Sample size		Heterogeneity (total)				MD or OR (95% CI)	*p* value (total)
		STPCNL	MTPCNL	Chi^2^	df	*I* ^2^ (%)	*p* value		
Initial SFR	3	543	699	4.84	2	59	0.09	0.80 (0.46, 1.38)	*p* = 0.42
3-month SFR	2	364	252	5.10	2	61	0.08	1.22 (0.38, 3.92)	*p* = 0.74
Auxiliary treatment	3	343	219	38.51	2	95	<0.00001	0.34 (0.03, 3.96)	*p* = 0.39
Hemoglobin drop	4	493	307	0.68	3	0	0.88	-0.47 (-0.68, -0.25)	*p* < 0.0001
Blood transfusion	5	657	759	3.6	4	0	0.46	0.48 (0.34, 0.67)	*p* < 0.0001
Operation time	2	323	199	7.74	1	87	0.005	-42.78 (-85.49, 0.07)	*p* = 0.05
Hospital stay	3	140	103	15.23	2	87	0.0005	-0.59 (-3.59, 2.41)	*p* = 0.70
Postoperative fever	2	136	49	0.01	1	0	0.9	0.86 (0.27, 2.78)	*p* = 0.8
Renal function	3	323	199	0.23	1	0	0.63	-0.02 (-0.06, 0.02)	*p* = 0.32
Urinary leakage	4	402	252	1.45	3	0	0.69	0.60 (0.19, 1.87)	*p* = 0.38
Urinary tract infection	3	99	73	0.02	1	0	0.89	0.85 (0.13, 5.45)	*p* = 0.87
Complications	4	480	636	0.97	3	0	0.81	0.74 (0.49, 1.12)	*p* = 0.15
Pulmonary complications	3	321	208	1.71	2	0	0.42	0.28 (0.09, 0.83)	*p* = 0.02
Nephrostomy time	2	323	199	3.23	1	69	0.07	0.94 (-2.39, 4.26)	*p* = 0.58

CI = confidence interval; MD = mean difference; RR = risk ratio.

## Data Availability

The datasets used and/or analyzed during the current study are available from the corresponding author on reasonable request.

## Abbreviations

AMSTAR: Assessing the methodological quality of systematic reviews
CI: Confidence interval
ESWL: Extracorporeal shockwave lithotripsy
LE: Level of evidence
MD: Mean difference
MTPCNL: Multiple tract percutaneous nephrolithotomy
OR: Odds ratio
PCNL: Standard percutaneous nephrolithotomy
PRISMA: Preferred Reporting Items for Systematic Reviews and Meta-Analyses
RCT: Randomized control trial
STPCNL: Single-tract percutaneous nephrolithotomy
SFR: Stone-free rate
UP: Upper pole
URL: Transurethral ureteroscope lithotripsy.
